# Nivolumab in Non-Small Cell Lung Cancer: Real World Long-Term Survival Results and Blood-Based Efficacy Biomarkers

**DOI:** 10.3389/fonc.2021.625668

**Published:** 2021-07-21

**Authors:** Sameh Daher, Yaacov R. Lawrence, Elizabeth Dudnik, Ekaterina Hanovich, Damien Urban, Nir Peled, Rossie Navon, Raya Leibowitz, Ariel Hammerman, Erez Battat, Teodor Gottfried, Amir Onn, Jair Bar

**Affiliations:** ^1^ Thoracic Cancer Unit, Institute of Oncology, Sheba Medical Center, Tel HaShomer, Israel; ^2^ Thoracic Cancer Unit, Davidoff Cancer Center, Rabin Medical Center, Petah Tikva, Israel; ^3^ Institute of Oncology, Shamir Medical Center, Zerifin, Israel; ^4^ Department of Pharmaceutical Technology Assessment, Clalit Health Services Headquarters, Tel Aviv, Israel

**Keywords:** nivolumab, NSCLC, long term survival, real life data, biomarker

## Abstract

**Objectives:**

We aimed to examine clinical data and baseline blood test results as potential predictive biomarkers for benefit from nivolumab, in advanced non-small cell lung cancer patients (NSCLC).

**Materials and Methods:**

A chart review was performed of 108 advanced NSCLC patients who commenced treatment with nivolumab between 2015-6 at three Israeli cancer centers, and for whom laboratory tests results were available. Data collected included sex, age, ECOG-PS, histology and number of previous lines of treatment. Baseline blood test results collected: absolute lymphocyte and neutrophil count (ANC), white blood cells (WBC), hemoglobin, platelets, albumin and lactate dehydrogenase (LDH). Neutrophil to Lymphocyte Ratio and ‘derived NLR’ (dNLR = (ANC/[WBC-ANC])) were calculated. Disease control at six months (DC6) was defined as any tumor shrinkage or stable disease during the first six months of nivolumab treatment. The association between clinical/laboratory variables and survival was tested with a Cox proportional hazard model. Data cut-off occurred in November 2019.

**Results:**

35 patients (32.4%) achieved DC6. Median overall survival (OS) of entire study population was 5.4 months. Four year survival rate was 16%. Achievement of DC6 strongly correlated with longer OS (HR 0.12, 95% C.I. 0.07-0.21, p<0.001). In univariate and multivariate analysis, dNLR, albumin and LDH correlated significantly with OS. No variables correlated significantly with DC6 in multivariate analysis. Based on albumin and LDH, we produced a score called CLAS (combined LDH and albumin score), including four prognostic groups of patients. Patients having low albumin and high LDH had the worst prognosis.

**Conclusion:**

In real-life setting, long-term efficacy of nivolumab in advanced line treatment of NSCLC is consistent with clinical trials. Response or stability of disease during first six months of treatment is associated with prolonged survival. We propose a novel score (CLAS) that may be useful for predicting outcome in nivolumab-treated NSCLC patients, but further validation is required.

## Introduction

Lung cancer is the leading cause of cancer -related death worldwide (1). Immune checkpoint inhibitors (ICI) for treatment of NSCLCdemonstrated superior survival of patients treated with the anti-PD-1 antibody nivolumab compared with docetaxel after failure of platinum-based chemotherapy (2–4). Pembrolizumab, another PD-1 inhibitor, and atezolizumab, an anti-PD-L1 inhibitor, have both also demonstrated significantly better overall survival (OS) in similarly designed trials, comparing each of them with 2^nd^-line docetaxel (5, 6).

Based on these trials, in 2015 nivolumab has received US Food and Drug Administration agency (FDA) and European Medicines Agency (EMA) approval for second-line treatment of NSCLC. Atezolizumab is similarly approved by FDA and EMA, while pembrolizumab is approved only for the treatment of patients with PD-L1 positive tumors (as included in the KEYNOTE 010 trial) (5). Response rate (RR) for each of these drugs in the 2^nd^ line setting is roughly 15-20%. Combined updated OS results from checkmate 017 and checkmate 057 show 13.4% OS rate at five years follow-up (3). In a landmark analysis of OS by response category at six months in checkmate 017 and checkmate 057, the OS rate at four years in nivolumab treated patients with CR/PR was 58%, and 19% for patients with SD at six months (3).

Efforts are being undertaken to identify biomarkers predicting response to immunotherapeutic agents (7). PD-L1 expression level and tumor mutational burden (TMB) are predictive for benefit from immunotherapy but are not always available and their predictive accuracy is limited (7–11). Additional molecular biomarkers are being investigated, such as STK11/LKB1 and KRAS (12–15). STK11/LKB1 genomic alterations were associated with shorter PFS and shorter OS in first line ICI treated NSCLC patients (12). On the other hand, several studies showed similar efficacy of ICI in KRAS mutant compared with KRAS wild type NSCLC patients (14, 15).

Numerous studies are onging, aiming to identify immune-related gene signatures correlating with clinical benefit from ICI. One of these assessed the utility of the 18-gene expression tumor inflammation signature in predicting ICI treatment outcome, and found it to predict clinical benefit of ICI in several tumors, including NSCLC (16). Another study reported predictability of selected gene signatures and genes for discriminating patients with durable clinical benefit from ICI from those with non durable benefit. These signatures and genes included the M1 signature, peripheral T cell signature, CD137 and PSMB9 mRNA expression (17). An additional study identified two signatures predicting outcome from ICI in chemotherapy-refractory advanced NSCLC patients, one reflecting the degree of immune infiltration and upregulation of interferon-gamma-induced genes, and a second reflected the epithelial-to-mesenchymal transition status (18).

Potential predictive biomarkers from the tumor microenvironment are being investigated in the PIONeeR study. The analysis for its first 100 patients, presented recently, suggested a predictive value for PDL1 positive cell density and density of cytotoxic T cells and immunosuppressive cells (regulatory T cells and myeloid-derived suppressor cells) in the tumor (19).

Levels of peripheral blood components, blood cells and various circulating molecules are an accessible potential non-invasive source of predictive biomarkers in NSCLC patients, and possibly relevant also for ICI-treated patients. Some of these biomarkers are validated prognostic markers for cancer in various scenarios. For example, high levels of serum lactate dehydrogenase (LDH) has been associated with poorer cancer-specific survival in several malignancies, including lung, colorectal and prostate cancer (20). Another example is albumin serum levels, known to be a robust predictor of survival in cancer patients in general and NSCLC patients in particular (21, 22). Interestingly, retrospective studies have demonstrated a correlation between high serum LDH levels at baseline and lower response rate (RR), and shorter (PFS) and OS in several types of cancer treated with anti-PD-1 and anti-PD-L1 antibodies (23–25). Hemoglobin levels can also serve as prognostic or potentially a predictive biomarker; several studies have demonstrated that anemia is linked to poorer prognosis in NSCLC patients (26, 27).

A number of studies investigated the peripheral blood cellular components as biomarkers of ICI efficacy. For ipilimumab-treated melanoma patients, improved OS and PFS were associated with low absolute neutrophil count, low neutrophil-to-lymphocyte ratio and high lymphocyte levels (28, 29). In a retrospective study including 607 pembrolizumab-treated melanoma patients, baseline elevated eosinophil count and elevated lymphocyte count were both associated with improved OS (30). Retrospective studies demonstrated elevated pre-treatment neutrophil-to-lymphocyte ratio (NLR) to be associated with shorter OS and PFS and with lower response rates in ICI-treated metastatic NSCLC patients (31–33). High NLR has been shown to be associated with decreased OS in a retrospective study analyzing the records of metastatic NSCLC patients enrolled on a number of phase I immunotherapy trials at the MD Anderson Cancer Center (32). However, NLR may not be relevant for all patients; a study demonstrated that in NSCLC, low NLR was correlated with favorable OS and PFS for patients with TMB > 10, while in patients with TMB ≤ 10, the differences between high and low NLR were not significant (33).

Conceivably, combining such biomarkers may derive a more accurate predictive or prognostic score. A group from Gustave Roussy analyzed data from a retrospective study of ICI *versus* chemotherapy for NSCLC patients and compiled a Lung Immune Prognostic Index (LIPI) (34). In this study, LIPI was correlated with worse outcomes for immunotherapy but not for chemotherapy. Other studies examining the applicability of LIPI in NSCLC and in solid tumors other than NSCLC reported results supporting its correlation with outcomes in ICI-treated patients (35, 36). In contrast, an exploratory pooled analysis of clinical studies of immunotherapy and targeted therapies for advanced NSCLC patients has shown LIPI to be associated with OS and PFS irrespective of treatment, emphasizing its prognostic rather than predictive role (37).

We report here real-world long-term survival results of NSCLC patients treated with nivolumab as a second or later treatment line in three Oncology centers, with a median follow-up of four years. We have comprehensively assessed clinical data and baseline blood levels of LDH, albumin and complete blood count results, as potential predictive biomarkers for benefit from nivolumab.

## Materials and Methods

### Study Conduct

This is a retrospective pharmacoepidemiological study, conducted, analyzed and reported according to REporting of studies Conducted using Observational Routinely collected health Data (RECORD) guidelines, the Strengthening the Reporting of OBservational studies in Epidemiology (STROBE), and ROCORD-pharmacoepidemiological research (RECORD-PE) guidelines (38–40). A chart review was performed of patients with advanced NSCLC that fit the study inclusion criteria.

### Patients

Inclusion criteria were age of 18 years and above, progression on first-line platinum-based chemotherapy, treatment at one of three participating Israeli cancer centers (Sheba Medical center (MC), Rabin MC and Shamir MC), administration of at least one cycle of nivolumab, with treatment commencing during 2015-2016. Exclusion criteria was lack of blood test results from the relevant time window (**Figure S1, Supplementary Data**).

### Data Collection

Data collected included sex, age, Eastern Cooperative Oncology Group performance status (ECOG-PS), histology and number of previous lines of treatment. Baseline blood test results collected included: absolute lymphocyte count (ALC), absolute neutrophil count (ANC), white blood cells (WBC), hemoglobin (HGB), platelets (PLT), albumin (ALB) and LDH. Baseline blood tests were defined as those performed prior to and within two weeks of the first nivolumab treatment. These parameters were categorized as high or low relative to the median of the study cohort, except for LDH, categorized as normal or above the upper limit of normal for the relevant clinical laboratory and albumin, categorized as normal or below the lower limit of normal for the relevant clinical laboratory. LIPI calculation is based on LDH greater than upper limit of normal (ULN) and ‘derived NLR’ (dNLR, calculated by: (ANC/[WBC-ANC])) > 3. Three risk groups were characterized: good: none of these factors, intermediate: one factor, poor: two factors. NLR, dNLR and LIPI score were calculated. In addition, CLAS (“Combined LDH and Albumin Score”) was defined as a suggested novel score and calculated as described in the results section.

Tumor assessments were performed by the treating physicians based on computerized tomography (CT) scans performed as part of the standard of care. CT scans were usually performed at two-three months intervals, at the treating physicians’ discretion. Two major outcomes have been assessed in our study: Disease control at six months (DC6) and OS. DC6 was defined as either present (any tumor shrinkage at any time during the first six months after initiating nivolumab treatment, or no change in tumors’ size for at least six months after starting nivolumab) or absent (any tumor growth or death at any time within the first six months).

OS was defined as time from initiation of nivolumab treatment until death, or censored at the last date patient was known to be alive.

### Statistics

Variables analyzed as categorial included sex and histology. In addition, LDH was redefined as a binary variable based upon whether the values being above or below the upper-limit of normal, and ALB was redefined as a binary variable based upon whether the values were above or below the lower-limit of normal. Variables analyzed as continuous included age, HGB, WBC, ANC, ALC and PLT. Variables analyzed as ordinal were ECOG-PS and number of previous lines.

The impact of covariates on survival was assessed using a Cox proportional hazard model. Multivariate analysis incorporated putative covariates found at univariate analysis to be significant at p < 0.1. Statistical comparisons were performed using Chi-squared test for categorical data and t-test for continuous variables. All p-values were two-sided, and p < 0.05 was considered statistically significant. The goodness-of-fit of different models were compared by examining pseudo-R^2^ values. Calculations were performed using Stata (version I/C 16.0, StataCorp). Data cut-off was at November 2019.

### Ethics

The study was approved by the institutional ethics committees of each of the participating centers. (Shamir Medical Center (MC): IRB #8993-11; Rabin Medical Center (MC): IRB #0391-14; Shamir MC: IRB #0062-17).

Investigators had complete access to the database of population included in the study. No patient identifying information was included in the study database.

## Results

### Patient Characteristics

A total of 108 patients treated with nivolumab were included in this study, mostly men (65%). Out of the study cohort, 35 patients (32.4%) had achieved DC6. More patients in the non-DC6 group had ECOG-PS of two or higher compared with DC group (p=0.030). The two groups were balanced in terms of other parameters (**Table 1**). Median follow-up was 48.7 months (IQR 47.4m – 53.0m).

**Table 1:** Baseline patient and disease characteristics.

Baseline patient/disease characteristics	All study cohort (108)	DC6 (35)	Non-DC6 (73)	P Value
Age – median (range), years	68 (40-96)	66 (40-85)	69 (43-96)	0.090
Sex – men – n (%)	70 (65%)	23 (66%)	47 (64%)	0.890
ECOG-PS – n (%)				0.030
0	4 (4%)	3 (9%)	1 (1%)	
1	46 (42%)	17 (48%)	29 (40%)	
2	40 (37%)	11 (31%)	29 (40%)	
3	17 (16%)	3 (9%)	14 (19%)	
Unknown	1 (1%)	1 (3%)	0 (0%)	
Histology – n (%)				0.220
Adenocarcinoma	64 (59%)	22 (63%)	42 (57%)	
Squamous cell carcinoma	29 (27%)	10 (28%)	19 (26%)	
NSCLC other*	15 (14%)	3 (9%)	12 (17%)	
No. of previous anticancer treatment lines$ - n (%)				0.440
0	12 (11%)	0 (0%)	12 (17%)	
1	79 (73%)	31 (88%)	48 (66%)	
2	10 (9%)	1 (3%)	9 (12%)	
≥3	7 (7%)	3 (9%)	4 (5%)	

DC6, Disease Control 6; ECOG-PS, Eastern Cooperative Oncology Group performance status; NSCLC, Non Small Cell Lung Carcinoma. *Other histologies included: large cell neuroendocrine (6), large cell undifferentiated (3), adenosquamous (1), NSCLC non-otherwise-specified (NOS; 5). $ Treatment lines for advanced disease prior to nivolumab treatment.

### Survival and DC6 Analysis

**Figure 1:**
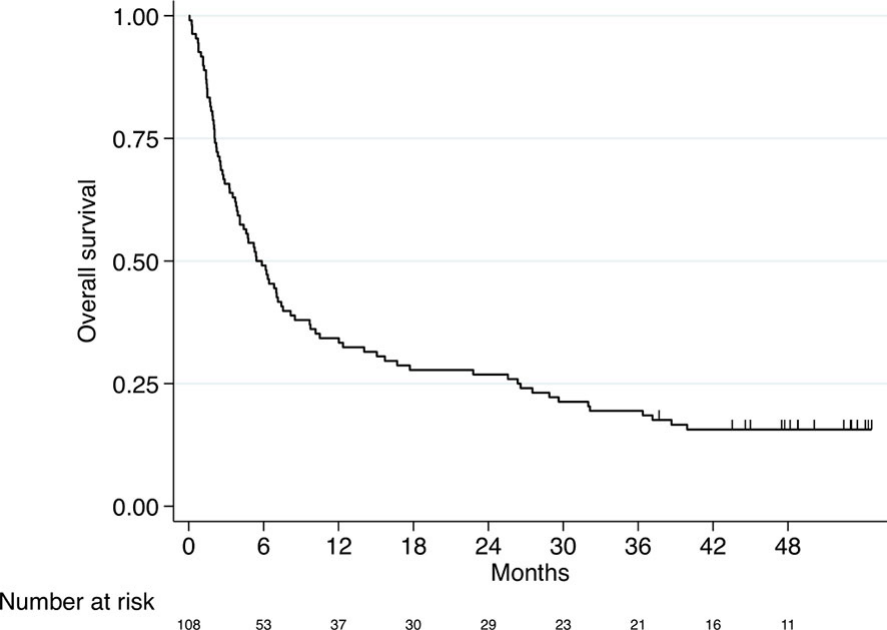
Kaplan–Meier curve for overall survival (entire study cohort, n=108). Median OS 5.4 months (95% CI 3.9-7.4).

The median OS of the entire study population was 5.4 months (95% CI 3.9-7.4, **Figure 1**).

Survival rates after one, two, three and four years were as follows: 34%, 27%, 19% and 16%, respectively.

**Figure 2:**
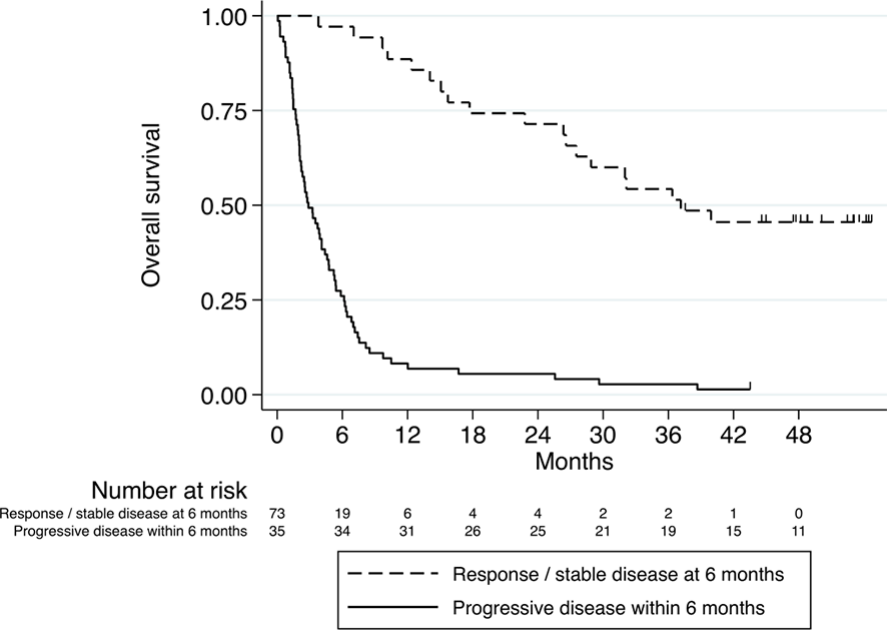
Kaplan–Meier curve for overall survival stratified according to DC6 (n=35) *vs.* non-DC6 (n=73) patients. HR 0.12, 95% confidence interval (CI) 0.07-0.21, p<0.001.

Of patients that have achieved DC6, 46% were alive at data cut-off, compared to 1% of patients that did not achieve DC6. In addition, Patients with DC6 demonstrated longer OS compared to patients without DC6 (HR 0.12, 95% confidence interval (CI) 0.07-0.21, p<0.001; **Figure 2**).

### Biomarkers

**Figure 3:**
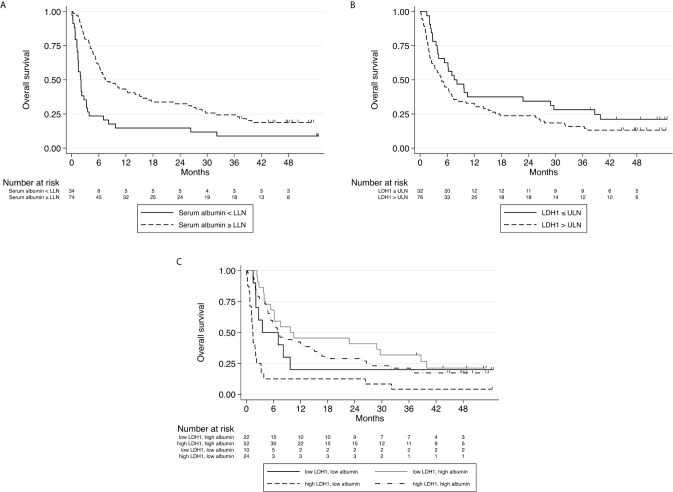
**(A)** Kaplan–Meier curve for overall survival stratified according to Albumin level at baseline (normal or below the lower limit of normal, n=108). **(B)** Kaplan–Meier curve for overall survival stratified according to LDH level at baseline (above upper limit of normal\below upper limit of normal, n=108). **(C)** Kaplan–Meier curves for overall survival stratified according to combined Albumin and LDH levels at baseline. **(A)** HR 0.33, 95% CI (0.20 - 0.52), p<0.001 **(B)** HR 1.15, 95% CI (1.06 - 1.25), p=0.001 **(C)** P<0.001.

We next tested the effects of the variables collected (demographic, hematological and biochemical) on OS. On univariate analysis, ECOG-PS and baseline values of WBC, ANC, NLR, dNLR, LDH and albumin were significantly correlated with OS. Due to overlap with dNLR, the variables WBC, ANC and NLR were not included in the multivariate analyses. On multivariate analysis, dNLR, albumin and LDH significantly correlated with OS (**Table 2** and **Figures 3A, B**), with high dNLR, low albumin and high LDH being adverse factors correlating with shorter survival. The statistical value of albumin and LDH was more significant than that of dNLR.

**Table 2:** Univariate and multivariate analysis for OS of the parameters investigated.

	Univariate Analysis		Multivariate Analysis	
	HR (95% C.I.)	P value	HR (95% C.I.)	P value
Age	1.02 (0.99 - 1.04)	0.148		
Sex	1.18 (0.76 - 1.83)	0.455		
**ECOG-PS**	**1.46 (1.12 - 1.89)**	**0.005**	1.31 (0.91 - 1.89)	0.141
Histology				
Adenocarcinoma	Comparator			
Squamous	1.02 (0.63 - 1.65)	0.933		
Other	1.47 (0.81 - 2.65)	0.207		
HGB	0.94 (0.83 - 1.05)	0.270		
**WBC**	**1.12 (1.06 - 1.18)**	**<0.001**		
**ANC**	**1.15 (1.09 - 1.22)**	**<0.001**		
ALC	0.97 (0.73 - 1.30)	0.860		
PLT	1.00 (1.00 - 1.00)	0.119		
**LDH**	**1.15 (1.06 - 1.25)**	**0.001**	**1.12 (1.03 - 1.22)**	**0.006**
**ALB**	**0.33 (0.20 - 0.52)**	**<0.001**	**0.36 (0.20 - 0.63)**	**<0.001**
**NLR**	**1.05 (1.02 - 1.07)**	**<0.001**		
**dNLR**	**1.18 (1.10 - 1.27)**	**<0.001**	**1.12 (1.01 - 1.24)**	**0.032**
Number of Previous Lines	0.85 (0.63 - 1.13)	0.260		

Statistically significant values are highlighted as bold. Due to overlap with dNLR, the variables WBC, ANC and NLR were not included in the multivariate analysis. HR, Hazard Ratio; C.I., Confidence Interval; ECOG-PS, Performance Status; HGB, Hemoglobin; WBC, White Blood Cells; ANC, Absolute Neutrophil Count; ALC, Absolute Lymphocyte Count; PLT, Platelets; LDH, Lactate Dehydrogenase; ALB, Albumin; NLR, Neutrophil to Lymphocyte Ratio; dNLR, derived Neutrophil to Lymphocyte Ratio. HR for LDH reflects LDH as calculated for each 100 international units per liter (IU/L). Number of patients with missing data: ECOG-PS: 1, HGB: 2, WBC: 3, ANC: 3, ALC: 4, PLT: 3, LDH: 28 and ALB: 26.

Regarding the binomial outcome DC6; age, ECOG-PS and baseline values of WBC, ANC, NLR, dNLR and albumin were all correlated with DC6 on univariate analysis. However, none of the investigated factors were found to be significant on multivariate analysis (**Table S1, Supplementary Data**).

**Figure 4:**
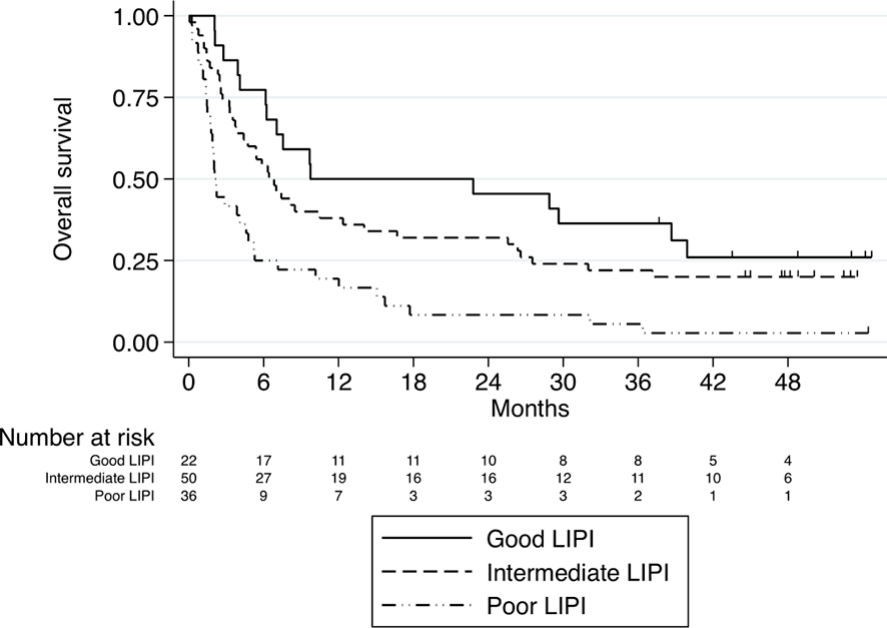
Kaplan–Meier curves for overall survival stratified according to LIPI score. Median OS for poor, intermediate and good LIPI was 2.1 months, 6.4 months, and 9.8 months, respectively, P<0.001.

We aimed to validate the prognostic role of LIPI in our cohort. Indeed, in our cohort LIPI was independently associated with OS (HR 1.8, 95% CI, 1.35-2.49, p < 0.001), leading to median OS for poor, intermediate and good LIPI of 2.1 months, 6.4 months, and 9.8 months, respectively (**Figure 4**). Therefore, our data provide further validation of the prognostic value of LIPI for nivolumab-treated advanced NSCLC patients.

Of the parameters examined in our study, baseline LDH and albumin levels were found to be most significantly correlated with survival. We attempted to produce a predictive score by combining these two variables, naming it ‘CLAS’. CLAS includes four prognostic groups of patients: high LDH + low albumin (CLAS 0), low LDH + low albumin (CLAS 1), high LDH + high albumin (CLAS 2), low LDH + high albumin (CLAS 3). Indeed, patients classified as CLAS 0 had the worst survival (p<0.001), while patients with CLAS 2 had better survival than patients with CLAS 1, suggesting that low albumin is worse than high LDH (**Figure 3C**).

## Discussion

Based on long-term (four years) follow up of our cohort, we report now real-world data of survival of a set of NSCLC patients treated with 2^nd^-line or higher ICI. In our study, the median OS of nivolumab-treated patients compares unfavorably with the median OS of 9.2 months and 12.2 months for patients with squamous and non-squamous NSCLC in CheckMate 017 and CheckMate 057 trials, respectively (2–4), as may be expected when comparing real-world data to clinical trial data. DC6 was found to be a strong predictor of OS in our cohort, consistent with published data analysis from these two checkmate trials (3). We also report baseline dNLR, ALB and LDH to be significantly and strongly associated with OS for these patients, with none of the examined variables found to be associated with DC6 on multivariate analysis. LIPI score was significantly correlated with survival as well, with poor LIPI group of patients having the worst outcomes, consistent with previous publications. Based on our data, we have produced a score we named CLAS combining ALB and LDH, which demonstrated strong correlation with survival, with low baseline albumin and high baseline LDH correlating with worst outcome. CLAS requires further validation in larger cohorts to clarify its contribution to the management of advanced NSCLC patients.

The relatively shorter median OS in our study may be attributed to the inclusion of patients with ECOG-PS 2/3 that constituted 52% (57 patients) of our cohort, while the randomized studies included only ECOG-PS 0/1 patients. Furthermore, 16% of the patients in our study were heavily pretreated, receiving nivolumab as a later than 2^nd^ treatment line. It should be noted that despite these poor prognostic characteristics, the long-term survival curve looks similar, with 16% long term survivors at data cut-off, consistent with data from the previously mentioned checkmate trials (2–4).

The two parameters outstanding from our data, which were included in the suggested ‘CLAS’ reflect tumor burden (LDH) and the patients’ nutritional status (ALB). The three variables that make up CLAS and LIPI score (LDH, albumin, dLNR) surprisingly performed better than ECOG-PS as predictors of OS. Despite the recognized validity of ECOG-PS as a strong prognostic factor, the inter-observer variability of this parameter may limit its usefulness in some cases (41). An important question relates to the predictive value of these parameters regarding chemotherapy treatment. Clinical and laboratory factors found to be prognostic in IO-treated NSCLC patients should be examined in parallel in large cohorts of chemotherapy-treated NSCLC patients, all treated with a similar type of chemotherapy. Such analyses would allow the assessment of the role of these factors as potentially predictive for outcome with IO treatments *versus* being general prognostic biomarkers.

A limitation of the study is its retrospective nature, with recognized drawbacks in terms of data accuracy and non-regular follow-up intervals. Furthermore, radiological response to treatment was based on investigators’ assessment and not on RECIST. In addition, the relevance of this cohort can be questioned, as most patients are now treated front-line with ICI (either as monotherapy or combined with chemotherapy). We raise here the possibility CLAS score could be applicable in the first-line setting as well, and examining our score in this setting is justified.

Another limitation is the relatively small size of the study, and the lack of a validation cohort to assess the accuracy of the suggested CLAS classifier. Such a validation cohort with equivalent prolonged follow up will be available in the future. However, our data of correlates of long-term survival allows insight into factors correlating with significant ICI efficacy, potentially with the postulated chance of cure. A larger data set is required for comparing the utility of LIPI and CLAS.

In conclusion, unlike median OS, the real life long-term efficacy of nivolumab in the advanced line setting in NSCLC is similar to data from published randomized trials. In addition, we propose a novel score we named ‘CLAS’ based on baseline albumin and LDH results as a potentially useful score for predicting outcome in nivolumab-treated NSCLC patients. The simple and unbiased measurement of these values adds to their apparent clinical applicability. This score needs further validation before any practical conclusions can be reached. Furthermore, we suggest categorization of patients on immunotherapy according to disease control after six months of treatment to be a simple and useful tool for similar studies. DC6 reflects the survival benefit of responding patients as well as of those with prolonged stability, and marks them as having a good chance for durable response and prolonged survival. Thus, this parameter should be further investigated as a surrogate factor for OS in ICI studies.

## Data Availability Statement

The raw data supporting the conclusions of this article will be made available by the authors, without undue reservation.

## Ethics Statement

The study was approved by the institutional ethics committees of each of the participating centers. (Sheba MC: IRB #8993-11; Rabin MC: IRB #0391-14; Shamir MC: IRB #0062-17). The studies involving human participants were reviewed and approved by Sheba Medical Center ethics committee. Written informed consent for participation was not required for this study in accordance with the national legislation and the institutional requirements.

## Author Contributions

SD: Conceptualization, Methodology, Investigation, Writing - original draft. YL: Formal analysis, Data curation, Writing - Review & Editing. ED: Conceptualization, Methodology, Investigation, Writing - Review & Editing. EH: Investigation, Writing - Review & Editing. DU: Conceptualization, Methodology, Writing - Review & Editing. NP: Conceptualization, Methodology, Writing - Review & Editing. RN: Resources, Writing - Review & Editing. RL: Conceptualization, Methodology, Writing - Review & Editing. AH: Resources, Data curation, Writing - Review & Editing. EB: Resources, Data curation, Writing - Review & Editing. TG: Resources, Formal analysis, Writing - Review & Editing. AO: Conceptualization, Methodology, Writing - Review & Editing. JB: Conceptualization, Methodology, Validation, Supervision, Project administration, Writing - Review & Editing. All authors contributed to the article and approved the submitted version.

## Funding

Nivolumab was provided to the majority of patients by Bristol-Myers Squibb (BMS) as part of a compassionate use program. BMS was not involved in collection, analysis, interpretation of data, manuscript drafting or submission.

## Conflict of Interest

The authors declare that the research was conducted in the absence of any commercial or financial relationships that could be construed as a potential conflict of interest.
