# Supplementary material for: Nivolumab in Non-Small Cell Lung Cancer: Real World Long-Term Survival Results and Blood-Based Efficacy Biomarkers
Source: Front Oncol. 2021 Jul 21;11:625668. doi: 10.3389/fonc.2021.625668 (PMC8335163; doi:10.3389/fonc.2021.625668)
Supplement: Supplementary file 2 [file Table_1.docx]

**Table S1**

Univariate and multivariate analysis for DC6 of the parameters investigated. Statistically significant values are highlighted as bold. Due to overlap with dNLR, the variables WBC, ANC and NLR were not included in the multivariate analysis. ECOG-PS: Performance Status. HGB: Hemoglobin. WBC: White Blood Cells. ANC: Absolute Neutrophil Count. ALC: Absolute Lymphocyte Count. PLT: Platelets. LDH: Lactate Dehydrogenase. ALB: Albumin. NLR: Neutrophil to Lymphocyte Ratio. dNLR: derived Neutrophil to Lymphocyte Ratio.

|  | Univariate Analysis | Multivariate Analysis |
| --- | --- | --- |
|  | P value | P value |
| **Age** | **0.091** | 0.227 |
| Sex | 0.893 |  |
| **ECOG-PS**  Histology  Adenocarcinoma  Squamous  Other | **0.030**  comparator  0.992  0.289 | 0.797 |
| HGB | 0.942 |  |
| **WBC** | **0.016** |  |
| **ANC** | **0.020** |  |
| ALC | 0.601 |  |
| PLT | 0.849 |  |
| LDH | 0.141 |  |
| **ALB** | **0.017** | 0.233 |
| **NLR** | **0.043** |  |
| **dNLR** | **0.054** | 0.063 |
| Number of Previous Lines | 0.442 |  |
